# Development and validation of an interpretable machine learning model for predicting cognitive impairment in patients with sepsis

**DOI:** 10.3389/fmed.2025.1753260

**Published:** 2026-01-21

**Authors:** Guisheng Liang, Mo Lu, Yurong Pan

**Affiliations:** 1 Department of Critical Care Medicine, Shenzhen Baoan Shiyan People’s Hospital, Shenzhen, China; 2 Department of Gynecology, Langxin Community Health Service Center, Shenzhen Baoan Shiyan People’s Hospital, Shenzhen, China

**Keywords:** cognitive impairment, interpretable machine learning, random forest, sepsis, SHapley Additive exPlanations

## Abstract

**Objective:**

Cognitive impairment is a common and debilitating complication after sepsis. This study aimed to develop and validate an interpretable machine learning (ML) model to predict post-sepsis cognitive impairment and identify key clinical risk factors.

**Methods:**

A retrospective cohort of 866 adult sepsis patients treated in our hospital between January 2020 and January 2025 was analyzed. Cognitive function was assessed 1–3 months after discharge using the Montreal Cognitive Assessment (MoCA), with scores < 26 indicating impairment. Key predictors were selected via least absolute shrinkage and selection operator (LASSO) regression and the Boruta algorithm. Five ML models—logistic regression, extreme gradient boosting (XGBoost), random forest (RF), k-nearest neighbors (KNN), and decision tree (DT)—were developed and evaluated using area under the curve (AUC), accuracy, F1-score. SHapley Additive exPlanations (SHAP) values were applied for interpretability.

**Results:**

Cognitive impairment occurred in 195 patients (22.5%). Seven variables were identified as key predictors of cognitive impairment, including age, years of education, septic shock, benzodiazepine use, acute physiology and chronic health evaluation II (APACHE II) score, sequential organ failure assessment (SOFA) score, and interleukin-10 (IL-10) level. The RF model performed best, with AUCs of 0.947 (training set) and 0.895 (validation set), showing good calibration and clinical utility. SHAP analysis showed that SOFA score had the greatest influence on cognitive impairment, followed by age, APACHE II score, IL-10, and years of education.

**Conclusion:**

Using SHAP analysis, the RF model provided clear insights into the key factors contributing to the model’s prediction of cognitive impairment after sepsis. The model not only achieved high predictive accuracy but also offered a transparent, data-driven tool to identify patients at elevated risk, potentially enabling timely interventions and tailored clinical management.

## 1 Introduction

Sepsis is a life-threatening syndrome characterized by dysregulated host responses to infection and remains a major global health challenge (1). Earlier global estimates suggested that sepsis affects approximately 31 million people annually and contributes to nearly 5 million deaths worldwide, underscoring its profound impact on population health (2). With improvements in critical care, a growing number of patients survive the acute phase of sepsis; however, many survivors experience long-term complications that substantially affect their quality of life (3). Among these sequelae, cognitive impairment has emerged as one of the most prevalent and debilitating outcomes. Studies have reported that a considerable proportion of sepsis survivors develop persistent deficits in memory, attention, executive function, or information processing, which may persist for months or even years after discharge (4, 5). These impairments not only hinder functional recovery and return to daily activities but also increase healthcare utilization, dependency, and long-term mortality. Despite its clinical significance, early recognition of cognitive impairment remains challenging because symptoms are often subtle and obscured by delirium, sedation, and other features of critical illness.

The development of cognitive impairment following sepsis is multifactorial. Proposed mechanisms include sustained systemic inflammation, blood-brain barrier disruption, microglial activation, neuronal injury induced by oxidative stress, and altered cerebral perfusion (6–8). At the clinical level, diverse factors such as disease severity, organ dysfunction patterns, metabolic disturbances, sedative exposure, and hemodynamic instability have all been implicated (5, 9, 10). However, the interaction among these variables is highly complex and non-linear, and traditional regression-based methods often fail to capture such intricate relationships. As a consequence, early and reliable prediction of cognitive impairment in sepsis is still limited, narrowing the potential to implement timely interventions and focused neuroprotective strategies.

Machine learning (ML) offers a powerful approach to address these limitations and has been applied to cognitive impairment following sepsis, both in clinical studies (11, 12) and animal models (13, 14). By leveraging large-scale, multidimensional clinical data, ML algorithms are capable of modeling non-linear interactions and improving predictive accuracy beyond conventional statistical techniques (15). Nevertheless, many ML models function as “black boxes,” producing predictions without revealing how input variables contribute to the outcome (16). In high-stakes clinical decision-making, this opacity raises concerns regarding accountability and interpretability, reducing clinicians’ willingness to adopt ML tools in practice. The emergence of interpretable ML provides a solution to this challenge (17, 18). Techniques such as SHapley additive exPlanations (SHAP) and model-agnostic interpretation frameworks allow visualization of feature contributions at both population and individual levels, enhancing transparency, reliability, and user acceptance (19). Applying interpretable ML to the prediction of cognitive impairment in sepsis may therefore improve early risk stratification, uncover clinically meaningful risk profiles, and support targeted strategies to prevent long-term neurological deterioration.

In this study, we aimed to develop and validate an interpretable ML model to predict the occurrence of cognitive impairment among patients with sepsis. By combining routinely available clinical variables with advanced interpretable ML techniques, we sought not only to optimize predictive performance but also to clarify the relative contribution of critical risk factors. This interpretable framework may assist clinicians in recognizing high-risk patients at an earlier stage and facilitate the implementation of strategies to mitigate long-term cognitive deterioration.

## 2 Materials and methods

### 2.1 Study cohort and participant selection

**FIGURE 1:**
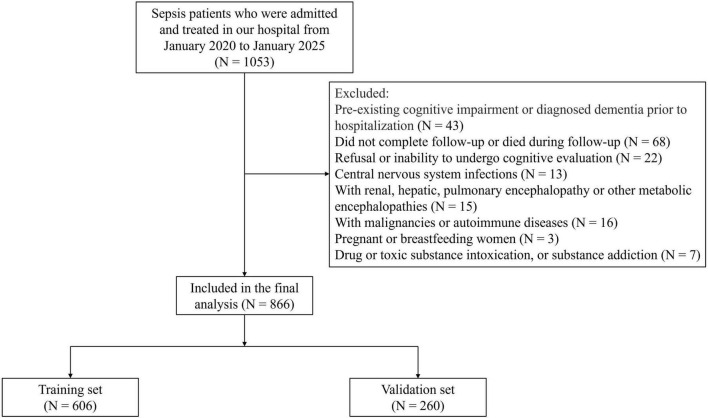
Study flowchart illustrating patient screening, enrollment, and grouping procedures.

This retrospective cohort study included adult patients diagnosed with sepsis and treated at our hospital between January 2020 and January 2025. Inclusion Criteria: (1) Diagnosed with sepsis according to the Sepsis-3 international consensus definition (20); (2) Age ≥ 18 years; (3) Patients who survived until discharge; (4) Patients with complete clinical data. Exclusion Criteria: (1) Pre-existing cognitive impairment or diagnosed dementia prior to hospitalization; (2) Did not complete follow-up or died during follow-up; (3) Refusal or inability to undergo cognitive evaluation; (4) Central nervous system infections, including meningitis or encephalitis; (5) With renal, hepatic, pulmonary encephalopathy or other metabolic encephalopathies; (6) With malignancies or autoimmune diseases; (7) Pregnant or breastfeeding women; (8) Drug or toxic substance intoxication, or substance addiction. After applying the exclusion criteria, 866 eligible patients were included in the analysis. To facilitate model development and internal validation, the entire cohort was randomly divided into a training set (70%) and a validation set (30%) using computerized random allocation. All feature selection and model training procedures were performed exclusively in the training set, while the validation set was used only for independent performance evaluation. A detailed flow of patient screening and enrollment was illustrated in Figure 1.

### 2.2 Follow-up and cognitive assessment

After hospital discharge, patients were followed for 3 months. Cognitive function was assessed using the Beijing version of the Montreal Cognitive Assessment (MoCA) (21). Evaluations were conducted at three predefined time points: approximately 1 month (30 ± 7 days), 2 months (60 ± 7 days), and 3 months (90 ± 14 days) after discharge. In accordance with scoring recommendations, 1 point was added to the total MoCA score for individuals with ≤ 12 years of education to correct for educational effects. Patients who had a MoCA score < 26 at any of the three follow-up assessments within 3 months were classified into the cognitive impairment group, whereas those with MoCA scores ≥ 26 at all assessments were assigned to the non-cognitive impairment group.

### 2.3 Data collection

Clinical data were collected retrospectively from the electronic medical records using a standardized case report form. Two trained investigators independently extracted and cross-checked all variables to ensure accuracy and completeness. Baseline demographic characteristics included gender, age, body mass index (BMI), marital status, years of education, and lifestyle factors such as smoking and drinking status. Comorbidities assessed at admission included hypertension, diabetes mellitus, and hyperlipidemia. Disease-related clinical variables comprised the presence of septic shock, the primary site of infection (respiratory, intra-abdominal, urinary, or other), and the use of critical care interventions, including mechanical ventilation, benzodiazepine administration, and vasopressor support. Severity of illness was quantified using the Acute Physiology and Chronic Health Evaluation II (APACHE II) and Sequential Organ Failure Assessment (SOFA) scores, calculated from the worst physiological and biochemical values recorded within the first 24 h after admission. Laboratory parameters were obtained from the initial blood samples collected during this same 24-h window and included white blood cell count (WBC), platelet count (PLT), procalcitonin (PCT), interleukin-6 (IL-6), interleukin-10 (IL-10), tumor necrosis factor-α (TNF-α), and serum uric acid (UA) levels.

The initial set of candidate variables was selected based on a combination of biological plausibility, evidence from prior literature, and availability in the electronic health record system. Demographic characteristics and established severity scores (SOFA and APACHE II) were included because they are widely recognized predictors of sepsis outcomes and neurological complications. Laboratory markers were chosen to reflect key pathophysiological domains relevant to post-sepsis cognitive impairment. Specifically, the cytokine panel (IL-6, IL-10, and TNF-α) was selected to capture systemic inflammatory and immune regulatory responses, which have been consistently implicated in sepsis-associated brain dysfunction (5, 7). Uric acid was included as a marker related to oxidative stress and metabolic disturbance, both of which may contribute to neuronal injury during severe systemic inflammation (22). Other commonly used sepsis biomarkers, such as lactate or C-reactive protein, as well as specific neuro-injury markers (e.g., S100B or neuron-specific enolase), were not consistently measured in this retrospective cohort and therefore could not be reliably incorporated into the initial analysis.

### 2.4 Model development and explainable machine learning

Prior to feature selection and model development, data preprocessing was performed. Continuous variables were standardized using z-score normalization (mean = 0, standard deviation = 1), while categorical variables were encoded as binary indicators. Because cases with incomplete demographic or laboratory information were excluded during cohort selection, no missing data imputation was required. To prevent information leakage, all preprocessing procedures were fitted using the training dataset and subsequently applied to the validation dataset.

To identify the most informative predictors of post-sepsis cognitive impairment, a two-stage feature selection strategy was adopted. First, the least absolute shrinkage and selection operator (LASSO) regression was applied to reduce dimensionality and eliminate redundant variables by imposing a penalty on the absolute size of coefficients. Subsequently, the Boruta algorithm was used to further confirm variable importance through iterative comparison with shadow features. The intersection of variables identified by both methods was selected as the final feature set for model construction.

Using these selected predictors, five ML algorithms were developed to construct prediction models: logistic regression, extreme gradient boosting (XGBoost), random forest (RF), k-nearest neighbors (KNN), and decision tree (DT). Hyperparameter tuning was performed within the training dataset for all ML models. Hyperparameters for all models were optimized using grid search combined with five-fold cross-validation. The training set was partitioned into five folds, with models iteratively trained on four folds and evaluated on the remaining fold. The combination of hyperparameters yielding the best mean cross-validation performance was selected for final model training. To prevent information leakage, the validation set was not involved in hyperparameter tuning or cross-validation and was used solely for independent performance evaluation. Model performance was comprehensively evaluated using multiple quantitative metrics, including area under the receiver operating characteristic (ROC) curve (AUC), accuracy, sensitivity, specificity, precision, and F1-score, etc. AUC served as the primary indicator of discriminative ability, while F1-score and accuracy were used as secondary evaluation metrics to further characterize overall performance. The model demonstrating the most favorable and stable results across these measures was selected as the final predictive model.

To assess the clinical utility and reliability of the final model, decision curve analysis (DCA), calibration curve analysis, and confusion matrix evaluation were conducted. Finally, to enhance interpretability and elucidate the contributions of individual predictors, SHAP analysis was employed to quantify both global and patient-level feature importance, thereby improving transparency and clinical interpretability of the model.

### 2.5 Statistical analysis

All statistical analyses were conducted using R software (version 4.3.2). Continuous variables were examined for normality using the Shapiro-Wilk test. Normally distributed variables were presented as mean ± standard deviation (SD) and compared using independent-samples *t*-tests. Non-normally distributed variables were expressed as median (interquartile range, IQR) and compared using the Mann-Whitney U test. Categorical variables were summarized as frequencies and percentages and compared using chi-square. Prior to model development, multicollinearity among predictors was examined by calculating the variance inflation factor (VIF) using the “car” package, and variables with VIF > 5 were considered to exhibit severe collinearity and excluded.

The “glmnet” package was used for LASSO regression, and the “Boruta” package was applied to identify important predictors via the Boruta algorithm. All ML models were developed and validated using the “tidymodels” package. Clinical utility was evaluated through DCA using the “rmda” package. Calibration performance was examined using calibration plots generated via the “rms” package. Confusion matrices were generated using “caret” package. Model interpretability was achieved using SHAP implemented through the “fastshap” and “shapviz” packages, which enabled quantification and visualization of global and individual-level predictor contributions. Statistical significance was defined as a two-tailed *P* < 0.05.

## 3 Results

### 3.1 Baseline characteristics of the study population

A total of 866 patients with sepsis were included in this study. The mean age of the cohort was 63.35 ± 9.37 years, and 53.9% were male. Overall, 195 patients (22.5%) developed cognitive impairment within 3 months after discharge. The dataset was randomly divided into a training set (*n* = 606) and a validation set (*n* = 260). As shown in Table 1, there were no significant differences between the two sets in age (63.23 ± 9.50 vs. 63.63 ± 9.08 years, *P* = 0.565), gender distribution (male: 54.1% vs. 53.5%, *P* = 0.857), or the overall incidence of cognitive impairment (21.9% vs. 23.8%, *P* = 0.540). Other demographic factors, lifestyle habits, comorbidities, sepsis-related clinical features, severity scores, and laboratory results also showed no significant differences between the two groups (all *P* > 0.05). These findings indicate that the training and validation sets were well balanced across baseline characteristics prior to model development.

**TABLE 1:** Comparison of baseline clinical profiles between the training and validation sets.

Variables	Total (*n* = 866)	Training set (*n* = 606)	Validation set (*n* = 260)	*χ ^2^/t/Z*	*P*
Gender				0.032	0.857
Male	467 (53.9%)	328 (54.1%)	139 (53.5%)		
Female	399 (46.1%)	278 (45.9%)	121 (46.5%)		
Age (years)	63.35 ± 9.37	63.23 ± 9.50	63.63 ± 9.08	0.575	0.565
BMI (kg/m^2^)	23.02 ± 2.28	23.09 ± 2.33	22.87 ± 2.16	1.301	0.194
Marital status				0.201	0.654
Coupled	688 (79.4%)	479 (79.0%)	209 (80.4%)		
Not coupled	178 (20.6%)	127 (21.0%)	51 (19.6%)		
Years of education	10.06 ± 2.56	10.14 ± 2.62	9.88 ± 2.41	1.371	0.171
Smoking				0.050	0.823
No	518 (59.8%)	361 (59.6%)	157 (60.4%)		
Yes	348 (40.2%)	245 (40.4%)	103 (39.6%)		
Drinking				0.041	0.840
No	703 (81.2%)	493 (81.4%)	210 (80.8%)		
Yes	163 (18.8%)	113 (18.7%)	50 (19.2%)		
Septic shock				0.240	0.624
No	721 (83.3%)	507 (83.7%)	214 (82.3%)		
Yes	145 (16.7%)	99 (16.3%)	46 (17.7%)		
Hypertension				0.142	0.706
No	561 (64.8%)	395 (65.2%)	166 (63.8%)		
Yes	305 (35.2%)	211 (34.8%)	94 (36.2%)		
Diabetes mellitus				0.235	0.628
No	685 (79.1%)	482 (79.5%)	203 (78.1%)		
Yes	181 (20.9%)	124 (20.5%)	57 (21.9%)		
Hyperlipidemia				0.107	0.743
No	586 (67.7%)	408 (67.3%)	178 (68.5%)		
Yes	280 (32.3%)	198 (32.7%)	82 (31.5%)		
Infection site				0.474	0.924
Respiratory tract	400 (46.2%)	283 (46.7%)	117 (45.0%)		
Intra-abdominal	177 (20.4%)	125 (20.6%)	52 (20.0%)		
Urinary tract	163 (18.8%)	111 (18.3%)	52 (20.0%)		
Other	126 (14.6%)	87 (14.4%)	39 (15.0%)		
Mechanical ventilation				0.022	0.882
No	207 (23.9%)	144 (23.8%)	63 (24.2%)		
Yes	659 (76.1%)	462 (76.2%)	197 (75.8%)		
Benzodiazepine use				1.278	0.258
No	731 (84.4%)	506 (83.5%)	225 (86.5%)		
Yes	135 (15.6%)	100 (16.5%)	35 (13.5%)		
Vasopressor use				0.226	0.635
No	556 (64.2%)	386 (63.7%)	170 (65.4%)		
Yes	310 (35.8%)	220 (36.3%)	90 (34.6%)		
APACHE II score (points)	17 (6, 30)	16 (6, 30)	19 (8, 29)	0.467	0.641
SOFA score (points)	7.37 ± 2.19	7.29 ± 2.20	7.55 ± 2.15	1.605	0.109
WBC (× 10^9^/L)	14.95 ± 3.40	14.99 ± 3.29	14.87 ± 3.65	0.476	0.634
PLT (× 10^9^/L)	158 (100, 213)	159 (101, 213)	154 (97, 211)	0.450	0.653
PCT (ng/mL)	8.19 ± 2.32	8.18 ± 2.38	8.22 ± 2.19	0.232	0.817
IL-6 (μg/L)	1.20 ± 0.40	1.19 ± 0.41	1.21 ± 0.37	0.677	0.499
IL-10 (μg/L)	0.73 ± 0.31	0.72 ± 0.31	0.75 ± 0.30	1.318	0.188
TNF-α (μg/L)	1.30 ± 0.40	1.31 ± 0.39	1.27 ± 0.42	1.352	0.177
UA (μmol/L)	355 (214, 439)	354 (215, 438)	357 (212, 441)	0.120	0.904
Cognitive impairment				0.376	0.540
No	671 (77.5%)	473 (78.1%)	198 (76.2%)		
Yes	195 (22.5%)	133 (21.9%)	62 (23.8%)		

BMI, body mass index; APACHE II, acute physiology and chronic health evaluation II; SOFA, sequential organ failure assessment; WBC, white blood cell count; PLT, platelet count; PCT, procalcitonin; IL-6, interleukin-6; IL-10, interleukin-10; TNF-α, tumor necrosis factor-alpha; UA, uric acid.

### 3.2 Baseline comparison by cognitive impairment status in the training set

Among the 606 patients in the training set, 133 (21.9%) developed cognitive impairment. As shown in Table 2, patients in the cognitive impairment group were significantly older than those without impairment and had fewer years of education (all *P* < 0.001). The incidence of septic shock was higher in the cognitive impairment group (*P* < 0.001), and benzodiazepine use was more common (*P* = 0.001). In addition, patients with cognitive impairment had markedly higher APACHE II scores and higher SOFA scores (all *P* < 0.001). Regarding laboratory indicators, IL-6 (*P* = 0.048) and IL-10 (*P* < 0.001) levels were significantly elevated in the cognitive impairment group, while other laboratory parameters, including WBC, PLT, PCT, TNF-α, and UA, showed no significant differences (all *P* > 0.05). No significant differences were found between the two groups in terms of gender, BMI, marital status, smoking or drinking habits, comorbidities, infection site, mechanical ventilation, or vasopressor use (all *P* > 0.05).

**TABLE 2:** Clinical and laboratory profiles of patients with and without cognitive impairment in the training set.

Variables	Cognitive impairment group (*n* = 133)	Non-cognitive impairment group (*n* = 473)	χ ^2^/*t*/*Z*	*P*
Gender			0.040	0.842
Male	73 (54.9%)	255 (53.9%)		
Female	60 (45.1%)	218 (46.1%)		
Age (years)	68.21 ± 8.62	61.83 ± 9.27	7.118	< 0.001
BMI (kg/m^2^)	22.88 ± 2.06	23.16 ± 2.41	1.220	0.223
Marital status			0.074	0.786
Coupled	104 (78.2%)	375 (79.3%)		
Not coupled	29 (21.8%)	98 (20.7%)		
Years of education	9.14 ± 2.25	10.43 ± 2.64	5.135	< 0.001
Smoking			0.024	0.878
No	80 (60.2%)	281 (59.6%)		
Yes	53 (39.8%)	192 (40.4%)		
Drinking			0.091	0.762
No	107 (80.5%)	386 (81.6%)		
Yes	26 (19.5%)	87 (18.4%)		
Septic shock			12.415	< 0.001
No	98 (73.7%)	409 (86.5%)		
Yes	35 (26.3%)	64 (13.5%)		
Hypertension			0.073	0.787
No	88 (66.2%)	307 (64.9%)		
Yes	45 (33.8%)	166 (35.1%)		
Diabetes mellitus			0.037	0.848
No	105 (78.9%)	377 (79.7%)		
Yes	28 (21.1%)	96 (20.3%)		
Hyperlipidemia			0.523	0.470
No	93 (69.9%)	315 (66.6%)		
Yes	40 (30.1%)	158 (33.4%)		
Infection site			0.118	0.990
Respiratory tract	63 (47.4%)	220 (46.5%)		
Intra-abdominal	28 (21.1%)	97 (20.5%)		
Urinary tract	24 (18.0%)	87 (18.4%)		
Other	18 (13.5%)	69 (14.6%)		
Mechanical ventilation			0.137	0.711
No	30 (22.6%)	114 (24.1%)		
Yes	103 (77.4%)	359 (75.9%)		
Benzodiazepine use			10.156	0.001
No	99 (74.4%)	407 (86.0%)		
Yes	34 (25.6%)	66 (14.0%)		
Vasopressor use			0.123	0.726
No	83 (62.4%)	303 (64.1%)		
Yes	50 (37.6%)	170 (35.9%)		
APACHE II score (points)	23 (12, 36)	13 (5, 28)	4.652	< 0.001
SOFA score (points)	9.15 ± 2.32	6.76 ± 1.85	12.409	< 0.001
WBC (× 10^9^/L)	15.17 ± 3.56	14.94 ± 3.21	0.712	0.477
PLT (× 10^9^/L)	152 (90, 212)	161 (106, 214)	1.129	0.259
PCT (ng/mL)	8.43 ± 2.18	8.11 ± 2.42	1.376	0.169
IL-6 (μg/L)	1.25 ± 0.45	1.17 ± 0.40	1.981	0.048
IL-10 (μg/L)	0.84 ± 0.37	0.69 ± 0.29	4.942	< 0.001
TNF-α (μg/L)	1.35 ± 0.42	1.30 ± 0.38	1.309	0.191
UA (μmol/L)	297 (169, 455)	355 (223, 436)	0.634	0.526

BMI, body mass index; APACHE II, acute physiology and chronic health evaluation II; SOFA, sequential organ failure assessment; WBC, white blood cell count; PLT, platelet count; PCT, procalcitonin; IL-6, interleukin-6; IL-10, interleukin-10; TNF-α, tumor necrosis factor-alpha; UA, uric acid.

### 3.3 Feature selection using a combined LASSO-Boruta approach

**FIGURE 2:**
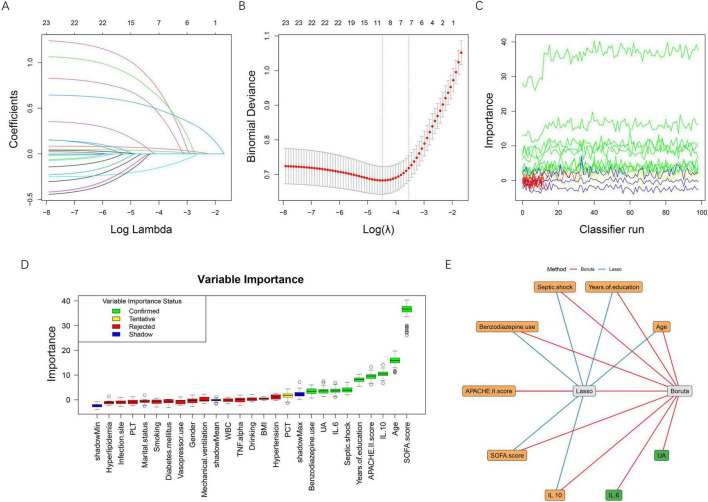
Feature selection using LASSO regression and the Boruta algorithm. **(A)** LASSO coefficient profiles of candidate variables plotted against log (λ). As the penalty increased, coefficients shrank toward zero, and variables with minimal contribution were gradually excluded. **(B)** Ten-fold cross-validation for selecting the optimal λ. The left dotted line indicates the λ value that yields the minimum binomial deviance (λ_min), while the right dotted line represents the most regularized model within one standard error of the minimum (λ_1se). The red dots represent mean binomial deviance, with error bars indicating ± 1se. **(C)** Feature importance trajectories produced by the Boruta algorithm over multiple classifier iterations. **(D)** Boxplots of Boruta variable importance. Confirmed features (green) and tentative features (yellow) demonstrate higher importance compared with shadow features (blue), whereas rejected features (red) fall below the threshold. **(E)** Integration of LASSO and Boruta results. Blue edges represent variables selected by LASSO, and red edges indicate variables confirmed by Boruta. The overlapping variables (orange nodes) were identified as the final feature set for model development. BMI, body mass index; APACHE II, acute physiology and chronic health evaluation II; SOFA, sequential organ failure assessment; WBC, white blood cell count; PLT, platelet count; PCT, procalcitonin; IL-6, interleukin-6; IL-10, interleukin-10; TNF-α, tumor necrosis factor-alpha; UA, uric acid.

A two-stage feature selection strategy combining LASSO regression and the Boruta algorithm was applied to identify the most informative predictors of post-sepsis cognitive impairment. In the LASSO analysis, the coefficient profiles progressively shrank toward zero with increasing penalty values (Figure 2A). Using 10-fold cross-validation, two candidate λ values were identified (Figure 2B). The λ corresponding to the minimum binomial deviance retained 10 variables, whereas the more parsimonious λ within one standard error (λ0.1se) retained 7 variables. To improve model simplicity and generalizability, we selected the λ0.1se model, which included the following seven features: age, years of education, septic shock, benzodiazepine use, APACHE II score, SOFA score, and IL-10.

The Boruta algorithm further evaluated the robustness of each variable through iterative comparisons with shadow features (Figures 2C–D). A total of nine predictors were ultimately confirmed as important, including age, years of education, septic shock, benzodiazepine use, APACHE II score, SOFA score, IL-6, IL-10, and uric acid, while all remaining variables were rejected. To ensure stability and clinical interpretability, the intersection of the two methods was taken as the final feature set. As illustrated in Figure 2E, seven variables overlapped between LASSO and Boruta: age, years of education, septic shock, benzodiazepine use, APACHE II score, SOFA score, and IL-10, which were subsequently used for model development.

### 3.4 Collinearity analysis

Before model construction, multicollinearity was assessed among the seven predictors retained after feature selection. The VIF values for each variable were as follows: age (1.015), years of education (1.024), septic shock (1.022), benzodiazepine use (1.013), APACHE II score (1.010), SOFA score (1.033), and IL-10 (1.031). All VIFs were well below the conventional threshold of 5, indicating that no severe multicollinearity was present among these predictors. Therefore, all variables were retained for subsequent ML model construction. For clarity, the definitions and value assignments of the variables were summarized in Table 3.

**TABLE 3:** Definitions and coding schemes for variables included in the analysis.

Variable type	Variable name	Value assignment
Independent	Age	Continuous
Independent	Years of education	Continuous
Independent	Septic shock	No = 0, Yes = 1
Independent	Benzodiazepine use	No = 0, Yes = 1
Independent	APACHE II score	Continuous
Independent	SOFA score	Continuous
Independent	IL-10	Continuous
Dependent	Cognitive impairment	No = 0, Yes = 1

APACHE II, acute physiology and chronic health evaluation II; SOFA, sequential organ failure assessment; IL-10, interleukin-10.

### 3.5 Model performance evaluation

**FIGURE 3:**
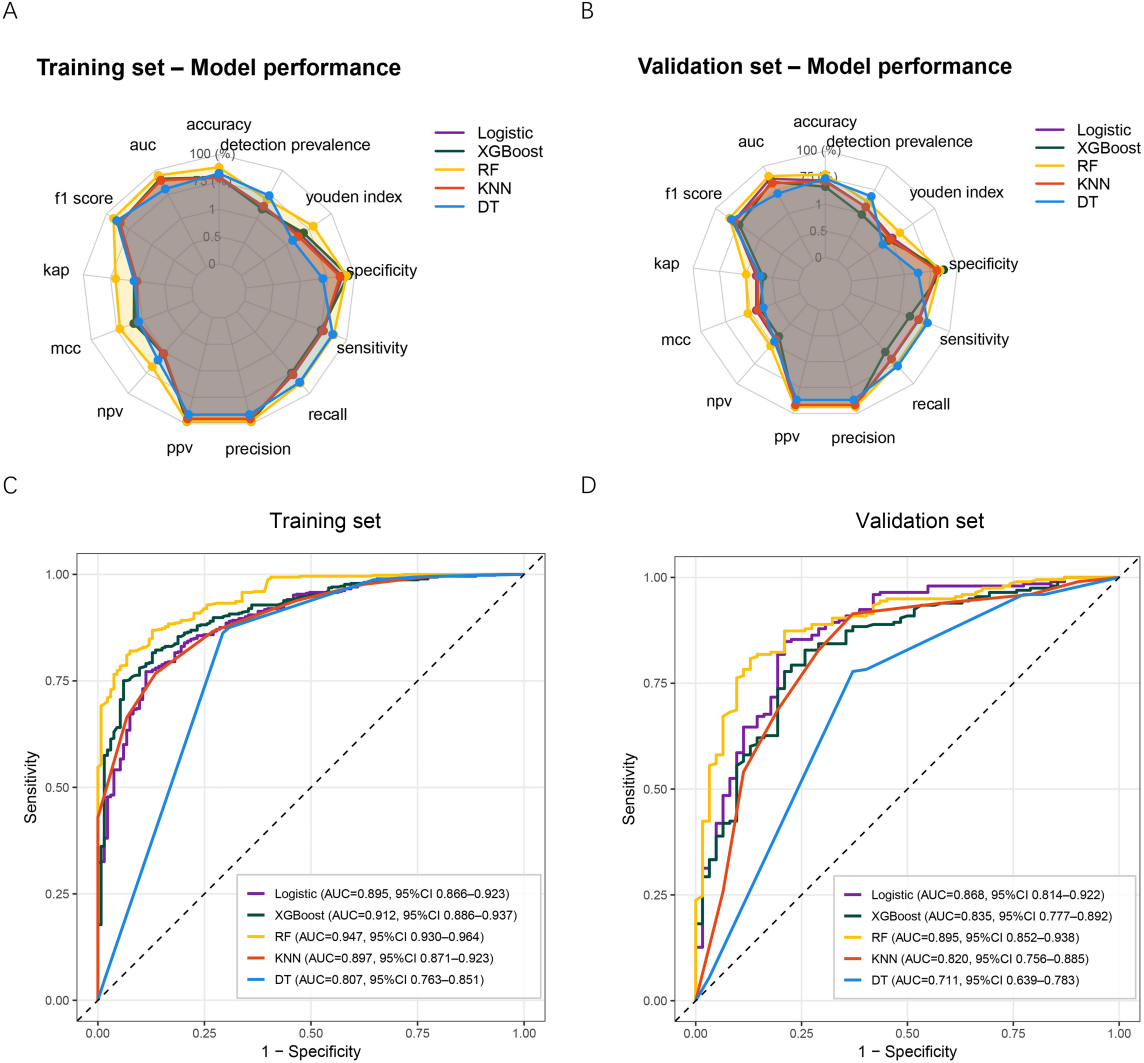
Performance of five ML models in predicting post-sepsis cognitive impairment. **(A)** Radar plot summarizing model performance across multiple evaluation metrics in the training set. **(B)** Radar plot summarizing model performance across the same metrics in the validation set. **(C)** ROC curves for the five models in the training set. **(D)** ROC curves for the five models in the validation set. ML, machine learning; XGBoost, extreme gradient boosting; RF, random forest; KNN, k-nearest neighbor; DT, decision tree; AUC, Area under the curve; MCC, Matthews correlation coefficient; NPV, negative predictive value; PPV, positive predictive value.

Five ML algorithms—logistic regression, XGBoost, RF, KNN, and DT—were constructed using the selected predictors. Model performance was assessed in both the training and validation sets using multiple evaluation metrics. As shown in Figure 3A, all models demonstrated acceptable performance in the training set, with RF, XGBoost, and logistic regression showing superior overall metrics, including accuracy, F1-score, precision, recall, specificity, and Youden index. Similar patterns were observed in the validation set (Figure 3B), where RF, XGBoost, and logistic regression maintained relatively stable and balanced performance, whereas DT showed weaker discrimination across several metrics.

ROC curve analysis further demonstrated the discriminative ability of each model. In the training set (Figure 3C), the RF model achieved the highest AUC (0.947, 95% CI: 0.930–0.964), followed by XGBoost (0.912, 95% CI: 0.886–0.937). The DT model yielded the lowest AUC (0.807, 95% CI: 0.763–0.851). In the validation set (Figure 3D), RF remained the best-performing model with an AUC of 0.895 (95% CI: 0.852–0.938), while DT again performed worst (AUC 0.711, 95% CI: 0.639–0.783). Taken together, the RF model demonstrated the most favorable and consistent predictive performance across both datasets and was therefore selected as the final model for subsequent clinical utility evaluation and explainability analysis.

### 3.6 Clinical utility and performance verification by calibration and confusion matrices in the RF model

**FIGURE 4:**
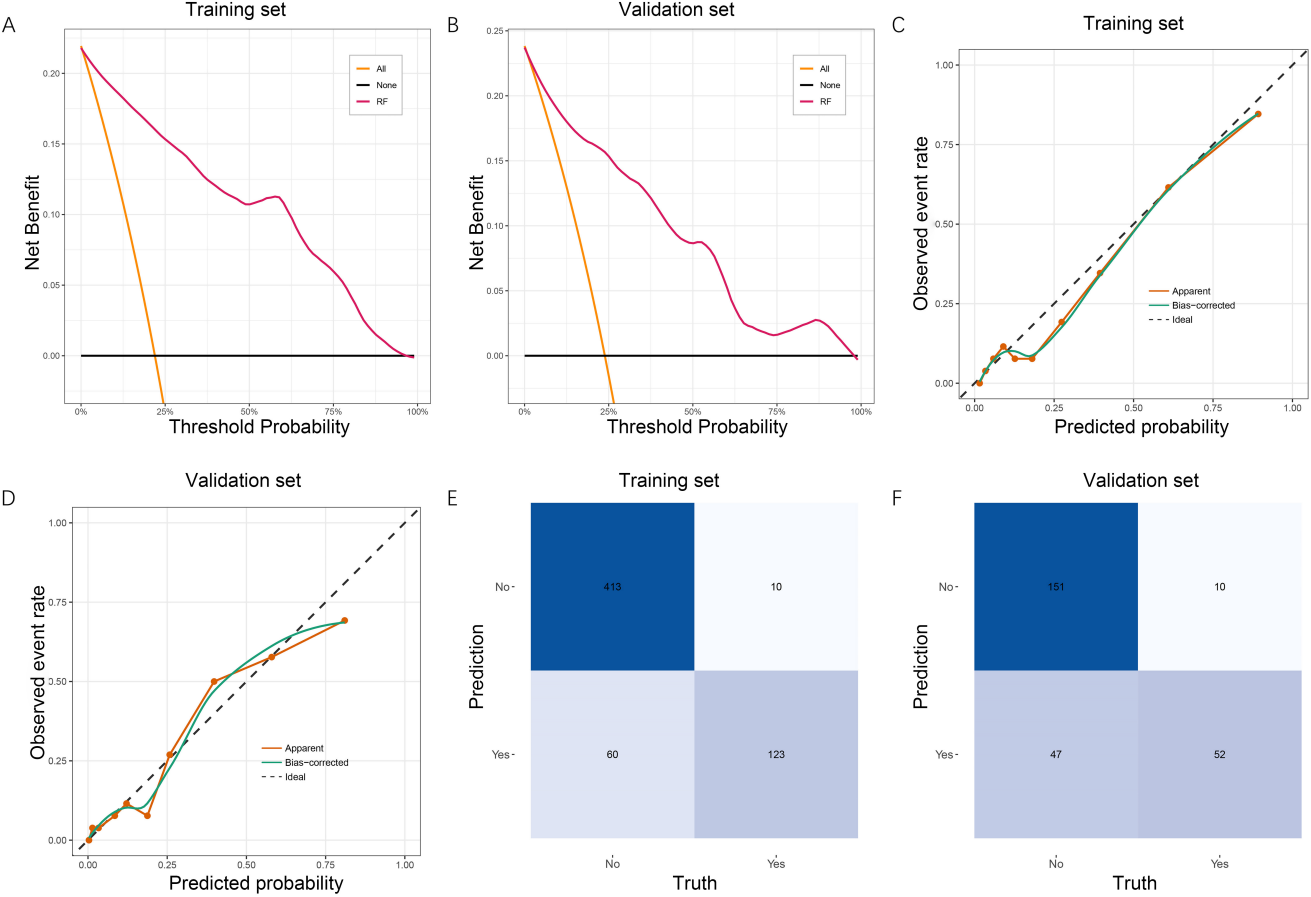
Clinical utility, calibration, and confusion matrix evaluation of the RF model. **(A)** DCA for the RF model in the training set, showing superior net benefit across a wide range of threshold probabilities compared with the treat-all and treat-none strategies. **(B)** DCA in the validation set, demonstrating consistent clinical usefulness of the RF model. **(C)** Calibration curve in the training set. The apparent and bias-corrected curves show good agreement between predicted and observed probabilities, indicating reliable model calibration. **(D)** Calibration curve in the validation set, confirming stable calibration performance. **(E)** Confusion matrix for the RF model in the training set, illustrating accurate classification of cognitive impairment and non-impairment cases. **(F)** Confusion matrix in the validation set, showing good generalizability of the classification performance. RF, random forest; DCA, decision curve analysis.

The clinical usefulness of the RF model was evaluated using DCA. In both the training set (Figure 4A) and validation set (Figure 4B), the RF model demonstrated a consistently higher net benefit across a wide range of threshold probabilities compared with the “treat-all” and “treat-none” strategies. This indicates that the RF model provides meaningful clinical value for guiding early identification of patients at high risk for post-sepsis cognitive impairment. Model calibration was then assessed using calibration curves. In the training cohort (Figure 4C), the apparent and bias-corrected curves closely followed the ideal reference line, suggesting good agreement between predicted and observed probabilities. A similar calibration pattern was observed in the validation set (Figure 4D), confirming good model reliability and stability. Confusion matrices further demonstrated the model’s classification performance. In the training set (Figure 4E), the RF model correctly identified 413 non-impaired and 123 impaired patients. In the validation set (Figure 4F), 151 non-impaired and 52 impaired patients were correctly classified. These results support the RF model’s balanced performance in both sensitivity and specificity. Collectively, the DCA, calibration analysis, and confusion matrices confirm that the RF model not only predicts cognitive impairment accurately but also offers substantial clinical benefit and generalizability.

### 3.7 Model interpretability based on SHAP analysis

**FIGURE 5:**
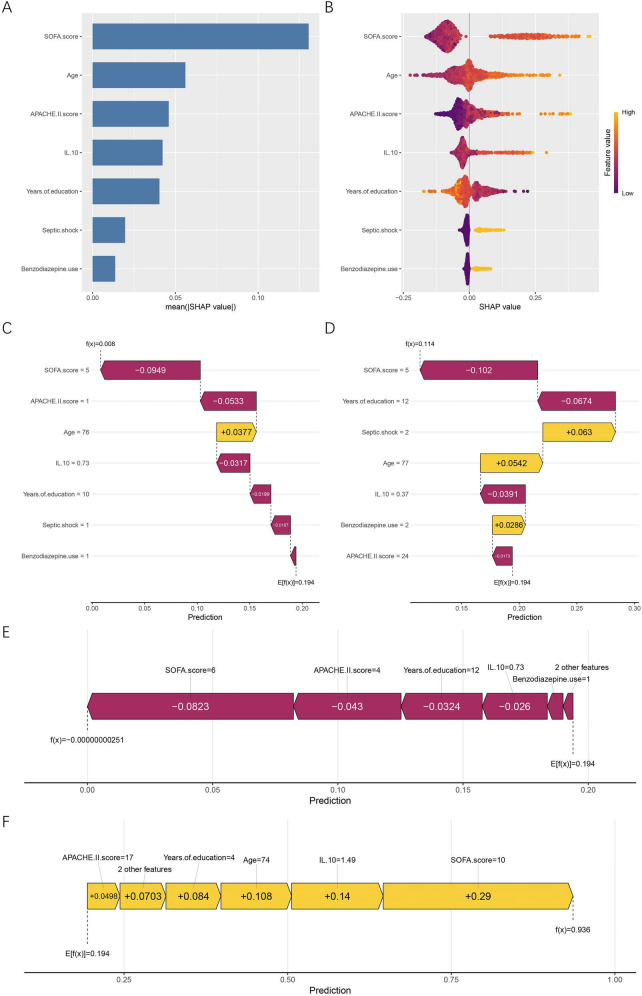
Global and individual-level SHAP interpretation of the RF model. **(A)** Mean absolute SHAP values showing the global importance ranking of all predictors. **(B)** SHAP summary (beeswarm) plot illustrating the direction and magnitude of feature influences. Feature values are color-coded from low (purple) to high (yellow). **(C)** SHAP force plot for the first patient in the dataset. **(D)** SHAP force plot for the last patient in the dataset. **(E)** SHAP force plot of a representative low-risk case. **(F)** SHAP force plot of a representative high-risk case. Yellow bars represent features that increase the predicted risk, whereas purple bars represent features that reduce it. E[f(x)] denotes the model’s baseline expected prediction, and f(x) refers to the individualized predicted probability for a given patient. SHAP, SHapley Additive exPlanations; APACHE II, acute physiology and chronic health evaluation II; SOFA, sequential organ failure assessment; IL-10, interleukin-10.

SHAP analysis was conducted to enhance interpretability of the RF model by quantifying both global and individual feature contributions, as well as characterizing non-linear relationships between predictors and cognitive impairment risk. At the global level, mean absolute SHAP values (Figure 5A) showed that the SOFA score was the most influential predictor, followed by age, APACHE II score, IL-10, and years of education. Septic shock and benzodiazepine use demonstrated smaller but meaningful contributions. The SHAP summary plot (Figure 5B) further illustrated the directional influence of features. Higher SOFA scores, older age, elevated APACHE II scores, and higher IL-10 levels increased the predicted probability of cognitive impairment, whereas greater years of education had a protective pattern that lowered predicted risk. For categorical variables, patients with septic shock (coded as 1) and those who received benzodiazepines (coded as 1) was associated with higher predicted probabilities within the model, compared with non-use (coded as 0). Individual SHAP force plots provided detailed interpretations for specific patients. For the first patient in the dataset (Figure 5C), lower SOFA and APACHE II scores drove the prediction downward and outweighed the modest positive influence of older age, resulting in an overall low predicted risk. For the last patient in the dataset (Figure 5D), advanced age and the presence of septic shock produced substantial positive contributions, shifting the prediction toward greater cognitive impairment risk. To illustrate extreme model behaviors, Figure 5E presents a typical low-risk case, characterized by low SOFA and APACHE II scores, higher years of education, and low IL-10 levels. In contrast, the high-risk case shown in Figure 5F demonstrates strong positive contributions from high SOFA scores, elevated IL-10, and older age, collectively pushing the prediction toward a near-certain risk.

**FIGURE 6:**
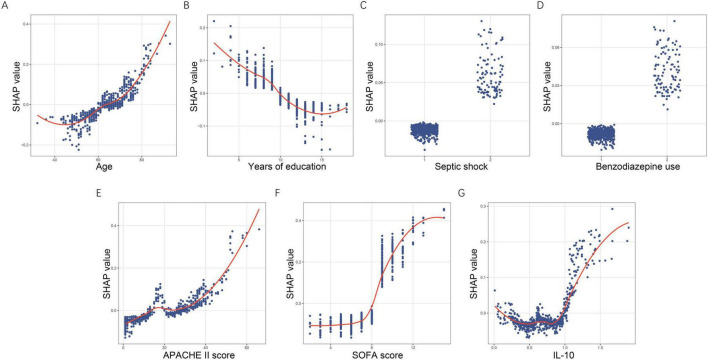
SHAP dependence plots demonstrating non-linear effects of key predictors. **(A)** Age. **(B)** Years of education. **(C)** Septic shock. **(D)** Benzodiazepine use. **(E)** APACHE II score. **(F)** SOFA score. **(G)** IL-10. Each dot corresponds to an individual patient, with the x-axis displaying the actual feature value and the y-axis representing its associated SHAP value. SHAP, SHapley Additive exPlanations; APACHE II, acute physiology and chronic health evaluation II; SOFA, sequential organ failure assessment; IL-10, interleukin-10.

SHAP dependence plots (Figures 6A–G) further revealed the non-linear relationships between predictors and their contributions to risk. SHAP values increased progressively with age, with a marked upward shift after approximately 65 years. Years of education showed a clear negative association, with higher educational attainment corresponding to lower predicted risk. For the binary predictors septic shock and benzodiazepine use, SHAP values remained near-zero when absent and shifted positively when present, supporting their role as risk-enhancing factors. APACHE II displayed an upward non-linear pattern, with sharply increasing contributions beyond scores of about 30. SOFA showed a sigmoidal pattern, with limited influence at low values and rapidly rising SHAP values above 7–8. IL-10 demonstrated a U-shaped pattern at lower concentrations followed by steep positive contributions once levels exceeded approximately 1.0 μg/L.

Together, these global, individual, and dependence-based SHAP analyses clarify which features exert the greatest influence, how variations in feature values shape predictions, and why individual patients are classified as high or low risk. These results substantially improve the transparency and clinical interpretability of the RF model.

## 4 Discussion

In this retrospective cohort study, we developed an interpretable ML framework to predict cognitive impairment among sepsis survivors and evaluated its performance using multiple complementary validation methods. The study identified seven key predictors using a combined LASSO-Boruta feature selection strategy, including age, years of education, septic shock, benzodiazepine use, APACHE II score, SOFA score, and IL-10. Among the five ML models constructed, the RF model achieved the most favorable and stable performance, with AUCs of 0.947 in the training set and 0.895 in the validation set. Calibration curves, DCA, and confusion matrices consistently confirmed the model’s stability, good clinical applicability, and balanced classification performance. SHAP-based interpretability analysis further clarified the global and individual contributions of the selected predictors. Among them, SOFA score emerged as the strongest contributor, followed by age, APACHE II score, IL-10, and years of education. These findings highlight the potential of an interpretable RF model to enhance early identification of sepsis survivors at high risk for cognitive impairment.

Although cognitive impairment is increasingly recognized as a major complication of sepsis, predictive studies remain scarce. Among the limited studies that have attempted to predict cognitive outcomes after sepsis, Zhu et al. evaluated three serum biomarkers and reported that their combined panel achieved an AUC of 0.887 in predicting persistent cognitive impairment in a cohort of 114 sepsis patients (23). Although the study offers initial insights into potential markers associated with cognitive outcomes after sepsis, its reliance on specialized biomarker assays, small sample size, and the absence of ML guided feature evaluation or interpretability limits its scalability and clinical practicality. In contrast, most other predictive efforts in the sepsis literature have focused on acute brain dysfunction rather than post-discharge cognitive outcomes. Jin et al. used MIMIC-IV data to develop a LASSO-based nomogram for predicting sepsis-associated encephalopathy (SAE), showing moderate discrimination. However, their model targets acute encephalopathy, not cognitive impairment (24). Similarly, Zhao et al. constructed a logistic-regression nomogram to predict acute SAE in elderly ICU patients, achieving an AUC of approximately 0.80, but their work likewise centers on in-hospital encephalopathy (25). Compared with these prior studies, our work provides substantial methodological and clinical advancements by focusing specifically on cognitive impairment, a clinically meaningful outcome that has been largely overlooked in previous predictive models. By leveraging routinely available clinical variables within a rigorous LASSO-Boruta framework and incorporating SHAP-based interpretability to characterize non-linear and individualized risk patterns, our model uses a RF algorithm that achieved excellent discriminative performance, with an AUC of 0.947 in the training cohort and 0.895 in the validation cohort, and offers a robust approach to anticipating cognitive impairment after sepsis.

The importance of the seven retained predictors is supported by existing biological and clinical evidence. Age is one of the strongest predictors of post-sepsis cognitive impairment. Older adults exhibit reduced cognitive reserve, diminished neuroplasticity, and greater vulnerability to inflammatory and hypoperfusion-related brain injury. Sepsis triggers systemic inflammation, microglial activation, oxidative stress, and blood-brain barrier dysfunction, all of which disproportionately impair aging neural circuits (26, 27). Overton et al. reported in a large population-based cohort that the prevalence of mild cognitive impairment rises substantially with advancing age, with older adults showing markedly higher rates of both mild and severe cognitive deficits across all cognitive impairment subtypes (28). Our findings similarly demonstrate that higher age is a key vulnerability factor for post-sepsis cognitive impairment. Individuals with higher educational attainment are believed to have greater cognitive reserve, which enables more efficient neural networks and resilience against brain injury from inflammation or hypoperfusion (29, 30). Concha-Cisternas et al. analyzed more than 2,000 Chilean adults aged ≥ 60 years and found that individuals with lower educational attainment had a markedly higher likelihood of cognitive impairment, with the risk increasing up to 4.53-fold in those with ≤ 8 years of education compared with those with higher education (31). This strong inverse association between education and cognitive performance supports the cognitive-reserve hypothesis and aligns with our finding that fewer years of education increase vulnerability to post-sepsis cognitive impairment. Patients who develop septic shock experience more pronounced circulatory and microvascular disturbances, which increase vulnerability to secondary brain injury through impaired cerebral perfusion and amplified inflammation (32). An MRI study by Götz et al. demonstrated that survivors of severe sepsis or septic shock exhibit structural alterations in key cognitive regions, including the hippocampus and amygdala (33). This evidence supports the strong association between shock physiology and cognitive impairment and aligns with our finding that septic shock significantly heightens the risk of post-sepsis cognitive decline. Benzodiazepine exposure has been reported to be associated with long-term cognitive outcomes in critically ill patients, often reflecting underlying acute brain dysfunction rather than a direct causal effect (34). Pandharipande et al. showed that lorazepam use independently increased the daily risk of transitioning into delirium in ICU patients (35). Stewart reviewed evidence from multiple peer-reviewed studies and reported that long-term benzodiazepine use is associated with impairments across several cognitive domains, with deficits that may persist even after discontinuation (36). The association between benzodiazepine use and post-sepsis cognitive impairment should be interpreted with caution. In critically ill patients, benzodiazepines are often administered in response to agitation, delirium, or severe physiological instability. As such, benzodiazepine exposure is subject to confounding by indication and may primarily reflect underlying acute brain dysfunction or illness severity rather than serving as an independent causal factor of long-term cognitive impairment. From a clinical perspective, benzodiazepine use in this context may act as a surrogate marker identifying patients who experienced more severe neurophysiological stress during the acute phase of sepsis. This interpretation is consistent with prior evidence linking sedative exposure, delirium, and subsequent cognitive decline. Therefore, our findings should not be interpreted as implying a direct neurotoxic effect of benzodiazepines *per se*, but rather as highlighting a high-risk clinical phenotype characterized by acute neurological vulnerability. Higher APACHE II and SOFA scores reflect more severe physiological derangement and multi-organ dysfunction during sepsis, factors that have been consistently linked to worse long-term neurological outcomes. Severe systemic inflammation, impaired perfusion, and metabolic instability captured by these scoring systems can exacerbate neuronal injury and hinder cognitive recovery (37). Iwashyna et al. reported that survivors of severe sepsis experienced a more than threefold increase in moderate-to-severe cognitive impairment compared with their presepsis baseline (4). A recent systematic review further showed that severe sepsis was associated with a significantly higher risk of dementia or cognitive impairment than milder forms of sepsis (10). These findings reinforce the concept that greater illness severity and organ failure burden markedly increase vulnerability to post-sepsis cognitive impairment, consistent with our observation that higher APACHE II and SOFA scores are key predictors of poor cognitive outcomes. It is important to interpret the contribution of severity-related scores within the context of a survivor-based cohort. In sepsis, patients with extremely high SOFA or APACHE II scores are at substantially increased risk of early mortality and are therefore less likely to survive to undergo post-discharge cognitive evaluation. As a result, the analyzed population represents a subset of sepsis survivors in whom the distribution of disease severity may be partially truncated. Within this framework, the strong influence of SOFA and APACHE II scores observed in our model suggests that, even among survivors, residual organ dysfunction and acute physiological derangement remain key determinants of subsequent cognitive impairment. Rather than reflecting mortality risk alone, these severity scores may capture the cumulative burden of systemic inflammation, hypoperfusion, and multi-organ dysfunction that predispose surviving patients to long-term neurocognitive sequelae. This distinction is clinically relevant, as it highlights that illness severity retains prognostic value beyond survival and may aid in identifying high-risk survivors who could benefit from early cognitive monitoring and intervention. IL-10 also emerged as a significant predictor in our model. Although IL-10 can exert anti-inflammatory or even neuroprotective effects in experimental models of acute neuroinflammation, elevated circulating IL-10 levels in sepsis primarily reflect severe immune dysregulation rather than direct protection (38, 39). Clinical studies have consistently shown that higher IL-10 concentrations correlate with greater organ failure, secondary infections, and increased mortality, indicating a state of sepsis-induced immunosuppression and more severe physiological derangement (40, 41). In this context, IL-10 likely serves as a biomarker of heightened systemic inflammation and immune paralysis—conditions known to amplify neuroinflammatory responses and hinder cognitive recovery. This interpretation aligns with our findings that higher IL-10 levels were associated with an increased risk of post-sepsis cognitive impairment.

Several methodological features of our study strengthen both the robustness of the model and its potential for real-world clinical application. By integrating a two-stage LASSO-Boruta feature selection strategy, we combined the strengths of penalized regression and iterative feature confirmation to minimize redundancy, reduce multicollinearity, and ensure the stability and clinical relevance of the retained predictors. The parallel evaluation of multiple ML algorithms further reduced model-specific bias, allowing an unbiased selection of the most reliable approach. Importantly, embedding SHAP-based interpretability addresses one of the major barriers to clinical adoption of ML—the opacity of prediction mechanisms—by providing transparent, patient-level explanations of how individual variables contribute to risk estimates. The methodological strengths of our model also translate into clinically actionable implications. Although septic shock, APACHE II, and SOFA scores primarily reflect underlying illness severity rather than directly adjustable interventions, the strong association of early physiological derangement with long-term cognitive impairment suggests that meticulous early resuscitation and organ-support strategies may indirectly contribute to neuroprotection. Together, these findings highlight several points in the acute-management trajectory where optimization may help mitigate the risk of post-sepsis cognitive decline. Although k-fold cross-validation is commonly used to enhance robustness in predictive modeling, a fixed training-validation split was intentionally adopted in this study. This design allowed us to retain an independent validation cohort for assessing discrimination, calibration, and clinical utility, as well as for illustrating SHAP-based interpretability on unseen data. Given that the primary objective of this work was to develop a clinically interpretable prediction framework rather than to optimize performance alone, preserving a hold-out validation set was considered appropriate. Nevertheless, we acknowledge that k-fold cross-validation may further improve generalizability, particularly in single-center settings. Future studies incorporating multicenter cohorts and cross-validation–based validation strategies are warranted to further evaluate model robustness and transportability. In addition to interpretability and predictive performance, the clinical applicability of ML models also depends on their generalizability across settings. Although the present model demonstrated strong discriminative performance and stability under internal validation, several issues related to generalizability should be considered. This study was conducted at a single center, and local clinical practices, patient characteristics, and sedation strategies may influence model behavior. Consequently, the current findings should be interpreted as reflecting performance within a similar clinical context rather than as universally generalizable estimates. Nevertheless, the use of conservative feature selection, independent validation splitting, and multiple complementary performance assessments was intended to reduce overfitting and enhance robustness. External validation using multicenter cohorts with standardized cognitive assessments will be essential to further evaluate model transportability and is a priority for future investigation. Several limitations should be acknowledged. First, this study included only sepsis survivors who were able to complete post-discharge cognitive assessments, which may introduce survivorship bias. Patients with extremely severe illness and early mortality were underrepresented, potentially limiting the generalizability of the findings to the entire sepsis population. Future multicenter studies are needed to validate the model across broader severity spectra. Second, this was a single-center retrospective study with internal validation only. Although multiple strategies were applied to reduce overfitting, including conservative feature selection and independent validation splitting, the absence of external validation limits the generalizability of the proposed model. Validation in multicenter cohorts with diverse clinical practices and patient populations is therefore warranted. Third, although MoCA is widely used for cognitive screening, it may not capture subtle deficits in specific domains such as processing speed or executive function. Incorporating comprehensive neuropsychological batteries or digital cognitive tools in future studies would yield more granular insights. Fourth, the follow-up duration in this study was limited to 3 months after hospital discharge, which may not fully capture the trajectory of post-sepsis cognitive impairment. Fifth, benzodiazepine use was treated as a binary variable due to limitations in the availability of detailed dosing and duration data. This simplified representation may not fully capture dose-dependent or time-dependent effects, and residual confounding by indication cannot be completely excluded. Finally, although a broad spectrum of clinical variables was incorporated, residual confounding cannot be completely excluded, particularly with regard to premorbid cognitive reserve, socioeconomic factors, and variations in sedation or hemodynamic management.

## 5 Conclusion

In summary, our study demonstrates that age, years of education, septic shock, benzodiazepine exposure, APACHE II and SOFA scores, and IL-10 levels are key features contributing to the model’s prediction of post-sepsis cognitive impairment. Among the ML models evaluated, the RF algorithm demonstrated the most robust and stable predictive performance. By integrating these variables into an interpretable RF framework, we were able to quantify the relative importance of each factor and enable fine-grained, individualized risk prediction to support personalized clinical management and early identification of high-risk patients. While the follow-up period was limited and external validation is needed, this model offers a promising tool for individualized risk stratification and may inform targeted strategies to mitigate neurocognitive sequelae in sepsis survivors. Future studies with extended follow-up and multicenter cohorts are warranted to refine predictive accuracy and elucidate the mechanisms underlying persistent cognitive impairment.

## Data Availability

The raw data supporting the conclusions of this article will be made available by the author, without undue reservation.
